# Measuring social exclusion in healthcare settings: a scoping review

**DOI:** 10.1186/s12939-018-0732-1

**Published:** 2018-02-02

**Authors:** Patrick O’Donnell, Diarmuid O’Donovan, Khalifa Elmusharaf

**Affiliations:** 1 Graduate Entry Medical School, University of Limerick, Limerick, Ireland; 2 School of Medicine, Clinical Science Institute, National University of Ireland, Room 342, Galway, Ireland

**Keywords:** Social exclusion, Social inclusion, Health inequality, Equity, Poverty, Measure, Instrument

## Abstract

**Background:**

Social exclusion is a concept that has been widely debated in recent years; a particular focus of the discussion has been its significance in relation to health. The meanings of the phrase “social exclusion”, and the closely associated term “social inclusion”, are contested in the literature. Both of these concepts are important in relation to health and the area of primary healthcare in particular. Thus, several tools for the measurement of social exclusion or social inclusion status in health care settings have been developed.

**Methods:**

A scoping review of the peer-reviewed and grey literature was conducted to examine tools developed since 2000 that measure social exclusion or social inclusion. We focused on those measurement tools developed for use with individual patients in healthcare settings. Efforts were made to obtain a copy of each of the original tools, and all relevant background literature. All tools retrieved were compared in tables, and the specific domains that were included in each measure were tabulated.

**Results:**

Twenty-two measurement tools were included in the final scoping review. The majority of these had been specifically developed for the measurement of social inclusion or social exclusion, but a small number were created for the measurement of other closely aligned concepts. The majority of the tools included were constructed for engaging with patients in mental health settings. The tools varied greatly in their design, the scoring systems and the ways they were administered. The domains covered by these tools varied widely and some of the tools were quite narrow in the areas of focus. A review of the definitions of both social inclusion and social exclusion also revealed the variations among the explanations of these complex concepts.

**Conclusions:**

There are several definitions of both social inclusion and social exclusion in use and they differ greatly in scope. While there are many tools that have been developed for measuring these concepts in healthcare settings, these do not have a primary healthcare focus. There is a need for the development of a tool for measuring social inclusion or social exclusion in primary healthcare settings.

**Electronic supplementary material:**

The online version of this article (10.1186/s12939-018-0732-1) contains supplementary material, which is available to authorized users.

## Background

The concept of “social exclusion” has become more prominent in discussions across many disciplines over the last number of decades. Politics, sociology, health and economics are just a few fields that have explored this complex idea and adapted it. There are many definitions of social exclusion but generally it describes the state of disadvantage faced by particular groups who are felt to be removed from mainstream society, and who cannot fully participate in normal life [1]. The phrase social exclusion originated in 1970s France when Socialist politicians began discussing the adversity faced by ‘les exclus’; a group of citizens who were not provided for by the state social security net [2]. The European Commission later introduced the term social exclusion into discussions alongside the term “poverty” for many programmes and initiatives from the early 1990s [3–5]; this culminated with 2010 being named as the European Year for Combating Poverty and Social Exclusion. Atkinson, when writing about the close relationship between social exclusion and poverty, said that an “analysis of social exclusion can broaden the discussion of well-being by considering dimensions beyond income poverty ... Being poor can lead to exclusion, but exclusion is more than just being poor, it is about participation” [3, 6]. Shaw and colleagues went further to explain that the term social exclusion can also encompass people “who may be stigmatized and marginalized, such as people with HIV/AIDS, who might not be considered in traditional analyses of economic deprivation” [7]. The government of the United Kingdom (UK) had also championed the idea of focusing on exclusion, establishing a specific Social Exclusion Unit (SEU) in 1997, which became part of the Office of the Deputy Prime Minister to drive this agenda across government departments and policymaking activity [3, 8, 9]. Many international bodies, such as the World Bank and the International Labour Organization, have also adopted the concept of social exclusion for use in their spheres of influence [10–12]. Commentators have discussed the apparent rise in popularity of this relatively new term social exclusion; one wrote that “conventional measures of poverty and deprivation were considered inadequate to capture the alienation, isolation or ‘exclusion’ from socially normative functioning” [13]. Others felt that this ‘new’ concept could also be seen as more acceptable politically and less stigmatising for those people who are actually affected by it [10, 14]. The widespread adoption of the term has been met with scepticism by others who have been critical of the move from focusing mainly on low levels of income as the primary cause of disadvantage; saying that now much of the blame for being socially excluded rests with the individual themselves, conveniently shifting the focus away from those with power and influence in society [14–16].

The closely related term “social inclusion” has also become popular in the related literature and in international policymaking. Charles Fraser gave a basic explanation of this term in 1999 when he said, “Social inclusion must come down to somewhere to live, something to do and someone to love. It’s as simple – and as complicated – as that” [17]. In Ireland, the term social inclusion has been adopted widely and appears frequently in policy documents across various sectors, particularly in health. The now disbanded Combat Poverty Agency defined social inclusion as “a series of positive actions to achieve equality of access to goods and services, to assist all individuals participate in their community and society, to encourage the contribution of all persons to social and cultural life and to be aware of, and to challenge all forms of discrimination”, clearly putting an onus on the government to be proactive in this regard [18].

The precise definitions of both social exclusion and social inclusion are highly contested. There is a growing body of literature that seeks to clarify the nuances of each term and the implications the various definitions have for corrective action and policymaking [4, 8, 10, 14, 19, 20]. Popay and colleagues reported that the definitions used to explain the concept of social exclusion generally fell into two broad categories: those that documented the many things that a person or group could be excluded from and those definitions that sought to explain a broader “relational” approach that looked more closely at the mechanisms and societal imbalances that led to and perpetuated social exclusion [8, 21]. Omtzigt concluded that that “definitions are caught between trying to provide an exhaustive list of everything the socially excluded is excluded from and listing the processes underlying the poverty and social exclusion” [22]. In recent years media reports and newspaper articles have begun to use these terms more frequently and without adequate explanation when reporting on a wide variety of societal problems and this seems only to add to the confusion around this terminology [23, 24].

### Why relate social exclusion to health?

Social exclusion is often mentioned as one of the social determinants of health. Actions to alleviate this state or the processes of exclusion are seen as crucial in addressing the health needs of all, and the health needs of marginalised groups in particular [7, 25]. A 2010 World Health Organization (WHO) report on poverty and social exclusion stated that these two factors were the “driving forces of health inequities for millions of people across the 53 Member States of the European Region” [26]. Groups that are commonly mentioned in the context of social exclusion and health include people who experience homelessness, people who are problem drug users, people who engage in sex work, Gypsies and Travellers and people with disabilities [27, 28]. Other sources mention numerous additional groups at risk of social exclusion: people who are unemployed, people who are migrants and refugees, people with mental health problems, women and children, older people, rural dwellers, people leaving institutions and single parent families [3, 29]. The seminal 2008 WHO World Health Report advised that making primary healthcare universal would ensure that “health systems contribute to health equity, social justice and the end of exclusion” [30]. This report and the subsequent 2010 WHO Europe report reinforced the significance of the role that health systems and primary healthcare have in addressing social exclusion and improving the health status of populations [26]. The authors summarised that “action to improve the health of disadvantaged populations should …. be grounded in a human rights approach to health and the values and principles of primary health care”, and highlighted the need to include “communities experiencing poverty and social exclusion in the design, implementation, monitoring and evaluation of policy and practice” [26].

Possibly the clearest discussion of the links between social exclusion and health took place in preparation for the 2008 WHO Commission on Social Determinants of Health. A subgroup of the Commission, called the Social Exclusion Knowledge Network (SEKN), was established in 2006 to investigate and report definitively on the relationship between these two concepts. The final SEKN report summarised that “social exclusion processes result in a continuum of inclusion/exclusion characterized by inequalities in; access to resources (means that can be used to meet human needs), capabilities (the relative power people have to utilize the resources available to them) and rights. This continuum results in health inequities. Social exclusion influences health directly through its manifestations in the health system and indirectly by affecting economic and other social inequalities that influence health. These inequalities contribute to social exclusion processes, creating a vicious circle” [8]. This detailed explanation clearly sets out that social exclusion, the problems that cause it, and those that derive from it, critically affects the health of individuals and populations.

This description links closely with the ethos of the United Nations Sustainable Development Goals (SDG’s), and goal number three in particular which is concerned with health and wellbeing across the life-course [31]. This SDG mentions the effective management of conditions such as HIV and substance abuse and the introduction of universal health coverage among other targets. This reflects the suggestion that improving the health status of such socially excluded groups may improve the health of the population as a whole. This also overlaps with the argument from some authors that health should be considered a human right and that a rights framework should be used to set appropriate standards and allocate responsibility for the improvement of the health status of certain groups in society [32].

### Why relate social exclusion to primary healthcare?

The field of primary healthcare is the ideal place to seek to document and analyse social exclusion in relation to health. Primary healthcare has wide population coverage in most countries. Primary healthcare services, such as general practice, work to alleviate many of the causes and ill effects of social exclusion on a daily basis – primary healthcare professionals understand that to cure or attempt to resolve the health problems of many of their vulnerable patients, they often need to find solutions to the exclusionary processes being experienced by those patients, as well as dealing with the actual medical issues. The UK’s Royal College of General Practitioners (RCGP) and National Health Service (NHS) have developed guidance specific to primary healthcare professionals and health service managers relating to the care of socially excluded groups [27, 29]. The advent of commissioning [where local health trusts in England and Wales plan and purchase services locally based on evidence of need] as a method of planning and funding community health services there has seen a focus on developing the case for service provision to groups traditionally described as socially excluded. Evidence is used to generate reports clearly outlining poor health outcomes for socially excluded groups when compared to the general population and then proposals are sought for possible interventions or adaptations to services in primary healthcare settings in order to attempt to close these health gaps [27, 28].

In 1995, Dr. Iona Heath wrote that general practitioners (GPs) often develop a deep understanding of the lives of their patients, and that GPs “see, every day, how society functions in a way which systematically undermines the health of its most vulnerable members” [33]. The conclusion that she and other authors have reached is that primary healthcare professionals, who work in such proximity to many socially excluded groups, have an onus to advocate and act on behalf of these patients [34–36]. For example, a GP may see and treat a person with community-acquired pneumonia using antibiotics and advise when to return if symptoms worsen. If that same person with the pneumonia is a person who injects drugs and who is rough sleeping, then the GPs advice and management may be different. He or she may attempt to secure hostel accommodation for that person, give information on where to obtain meals, help to locate an addiction support worker for the patient, discuss the safe storage of medications and possibly plan an early clinical review of the patient. Existing measures of success in primary healthcare interventions with marginalised patients are generally limited to the traditional disease mortality and morbidity outcomes; but there is the possibility that these do not capture the essence of life and health as a socially-excluded person. Bearing these factors in mind, we are seeking to discover if the degree of social exclusion a person is experiencing – in all its complexity and with the ambiguity associated with the terminology – could be an appropriate measure for use in primary healthcare settings.

This scoping review was therefore developed to address the following specific questions: how are social exclusion and social inclusion defined in relation to health, and how are social exclusion and social inclusion measured at the individual level in healthcare settings. Measuring the degree of social exclusion of a person attending a healthcare service could allow their status to be monitored over time, and potentially show that certain healthcare interventions reduce social exclusion. This may demonstrate that health policies and health system interventions aimed at marginalised and socially excluded groups have tangible benefits. A scoping review allows us to summarise the characteristics of measures of social exclusion and social inclusion that have previously been developed, and highlight any gaps in the extant evidence. Scoping reviews do not typically involve detailed critical appraisal of the included work, thereby allowing a variety of both peer-reviewed and grey literature to be included.

## Methods

This scoping review of the literature was conducted by applying the methodological framework set out by Levac and colleagues, which was based on prior work by Arksey and O’Malley [37, 38]. The steps include (i) identifying research questions, (ii) identifying all relevant studies, (iii) selecting significant studies, (iv) charting the relevant data, and then (v) summarising and reporting the results.

### Identifying relevant studies

To find publications related to these research questions we searched electronic databases, reference lists and key websites for both peer-reviewed papers and grey literature. The databases accessed included Academic Search Complete, CINAHL, EconLit, Medline, Social Sciences Full Text, Web of Science, EMBASE and Psyc-INFO. The search strategy for these databases included three rows of search terms to be applied to the titles and abstracts of publications. These were Row 1: “social incl*” OR “social excl*” OR “social marginal*”, Row 2: health* and Row 3: measure* OR frame* OR index OR indic* OR monitor* OR scale OR tool OR instrument. The specific websites that were searched included those of the United Nations Development Programme, the World Bank and the WHO.

### Selecting the studies

For this review, final inclusion and exclusion criteria were developed as the searching and exploration of the resulting papers took place [37]. Criteria included work published in English between January 2000 and January 2017 from any country. Publications to be included were peer-reviewed research, published reports, editorials, commentaries and PhD theses. Publications for inclusion had to relate primarily to social exclusion or social inclusion and their measurement in relation to health. For the exclusion criteria documents such as conference abstracts and book reviews, publications not relating primarily to social exclusion or social inclusion and its measurement in relation to health and publications reporting on biological or physiological responses to exclusion were omitted. The lead author was responsible for screening the titles and abstracts of all document using the agreed inclusion and exclusion criteria. The co-authors were then consulted at regular intervals during the review process to discuss the emerging results, and to resolve any issues arising in the search process. For the results, we focused on measurement tools looking at social inclusion or social exclusion at the individual patient level, and their supporting publications.

### Charting the data and reporting the results

The details of each of the measurement tools included in the final scoping review are displayed in Table 1 under the headings of (i) name of the tool, (ii) whether it mentions primarily social exclusion or social inclusion, (iii) the target population group for the tool, (iv) the purpose of the tool, (v) a brief background on the tool, (vi) the number of items included in the tool, (vii) how the tool reports results, and (viii) how it is administered to participants. A number of review papers were discovered during the searches and these are noted on the table in Additional file 1. A sample of the definitions of social exclusion and social inclusion referenced in the background papers for each of the twenty-two tools is also included in Tables 3 and 4.

**Table 1:** Measurement Tools

Full Name [and associated references]	Name	Social Exclusion (SE) / Social Inclusion (SI)	Target Group	Purpose	Background	No of Items	Scoring	Administration
Activity and Participation Questionnaire [42, 43, 64]	APQ-6		patients in mental health settings	to foster discussion on recovery between patients in mental health settings and clinicians	developed from concepts of Australian Bureau of Statistics Labour Force Surveys and Census	6	measures hours of participation	self-reported, telephone or face to face
Australian Community Participation Questionnaire [42, 43, 65]	ACPQ		general population, patients in mental health settings	to measure community participation in people with mental health issues, including those in rural and remote locations, not for clinical use	developed specifically	15 (short version), 30 (long version)	Likert scale	self-reported
Based On ‘The Human Givens’ [40, 41]	HG		patients in mental health settings					
Community Integration Measure / Questionnaire [57, 66, 67]	CIM / CIQ		patients with traumatic brain injury, patients in mental health settings	to measure belonging and participation in community	based on qualitative research with patients with brain injury	10	Likert scale	self-reported
Composite Measure Of Social Inclusion [42, 43, 68, 69]	CMSI	SI	patients in mental health settings	to measure social inclusion as an outcome of psychosocial rehabilitation programs	based on aspects of the Socially Valued Role Classification Scale [70] and the Community Integration Questionnaire [67]	75	classification table including hours of participation and supports needed	two separate face to face interviews
EMILIA Project Questionnaire [42, 43, 56]	EPQ	SI/SE	patients in mental health settings	to examine changes in SE/SI of mental health service users who took part in educational programme, for use in clinical setting and for research	developed specifically	10	qualitative only, answers analysed thematically	self-reported
Evaluating Social Inclusion Questionnaire [71]	ESIQ	SI	patients in mental health settings	to evaluate patient’s sense of social inclusion, especially when they move from supported settings out into the community.	focus on service user/patient input into design and reporting of tool	18	Likert scale	semi structured interview
Inclusion Web [42, 43, 72]	IW	SI	patients in mental health settings	for use in discussion between patient and clinician to foster collaboration and to improve social inclusion	developed specifically	18	counting no of activities, people and places engaged with	patient and HCP discussion, visual ‘map’ developed
Living in the Community Questionnaire [42, 73]	LCQ	SI	patients in mental health settings	to monitor the social inclusion of patients in mental health settings; including vocational and community activity, housing status and access to a GP, for use in clinical setting	developed by the Australian Mental Health Outcomes and Classification Network, based on APQ 6 tool	33	Likert scale	self-reported
Mental Health Recovery Star [74–76]	MHRS	SI	patients in mental health settings	to assess patients recovery from mental illness, key workers to map and plan change	based on HOS below, but then academic literature and published mental health h service user accounts also influence	10	scale 1–10 for each domain	patient and key worker discussion, visual ‘map’ developed, repeated over time
Multidimensional Social Inclusion [36]	MSI	SI	patients in mental health settings	tool to prompt discussion between patients in mental health settings and clinicians on social inclusion	tool developed meant to facilitate discussion in clinical setting, based on theory of Pinfold [77]	4	not scored	discussion aid
Homeless Outcomes Star [78–80]	HOS		homeless people	to measure change across ten domains of life of homeless person, focus on self-reliance, key workers use to map and plan change	developed by staff and clients of homeless organisations, ‘bottom-up process’	10	scale 1–10 for each domain	patient and key worker discussion, visual ‘map’ developed, repeated over time
Participation Scale [81]	PS	SI	people with disability	to evaluate rehabilitation, social inclusion and stigma reduction programmes, available in seven languages, for research or clinical assessment of progress	based partly on participation domains of International Classification of Functioning, Disability and Health [82]	13 (short version), 18 (long version)	overall score calculated	interview by staff
Social and Community Opportunities Profile [41–43, 57, 83]	SCOPE	SI	patients in mental health settings, general population	to assess social inclusion using objective and subjective measures	based on review of existing SI measures and literature, concept mapping exercises conducted with different groups incl mental health service users, professionals and general population	48 (short version), 121 (long version)	Likert scale and other measures	self-reported or interview
Social and Community Opportunities Profile – Chinese Version [44, 84–87]	SCOPE-C	SI	patients in mental health settings, people who are migrants	to assess social inclusion with objective and subjective factors with people in cultural context	Chinese version of SCOPE, modified based on local research and translated for use in Hong Kong	56	Likert scale and other measures	self-reported
Social Inclusion Questionnaire [42, 43, 47]	SIQ	SI	patients in mental health settings	to estimate the level of social inclusion of patients in mental health settings attending a day hospital service, for use in clinical setting	looked at existing measures and then developed own over repeated engagement with service users and staff	23	Likert scale and traffic light system from Bates 2002 to report level of SI	self-reported
Social Inclusion Questionnaire User Experience [45, 57, 88]	SInQUE	SI	patients in mental health settings	to assess the extent to which people with severe mental illness are socially included; looking back at time prior to hospital admission, and at current situation	based on the Poverty and Social Exclusion Survey [89] with added questions on political engagement	97	score given for each domain, then total score calculated; higher score means greater SI	structured interview
Social Inclusion Scale / Social Inclusion Measure [42, 43, 57, 69, 90–92]	SIS / SIM	SI	patients in mental health settings	to assess how participation in arts programmes affects social inclusion	created specifically, but based on literature reviews, website searches, contacting experts and UK National Labour Force Survey questions	22	Likert scale	self-reported
Social Integration Survey [57, 93]	SIS		patients in mental health settings	to measure social functioning for patients with schizophrenia from patient and informant perspective	based on literature, expert advice and patient feedback	62		self-reported
Staff Survey of Social Inclusion [42, 43, 94]	SSSI	SI	patients in mental health settings	for mental health staff to estimate social inclusion of patients using traffic light system, for use in clinical setting	developed specifically, but based on Bates and Butlers ‘Life Domains’ [95]	10	staff report time patient has spent on activity over one week	staff complete the measure
Support Needs Questionnaire [96]	SNQ	SI	patients in mental health settings	to measure social inclusion and recovery of people with significant mental health problems, for use in clinical setting	derived from Social Role Valorisation theory [97], developed in conjunction with patients	8	Likert scale	face to face
Vulnerability Assessment Tool [98, 99]	VAT		homeless people	used to measure homeless persons vulnerability and needs in order to allocate services	developed by staff in homeless services to assess need and allocate resources	10	scale 1–5 for each domain, overall score calculated	person and key worker

## Results

### Flow diagram

**Fig. 1:**
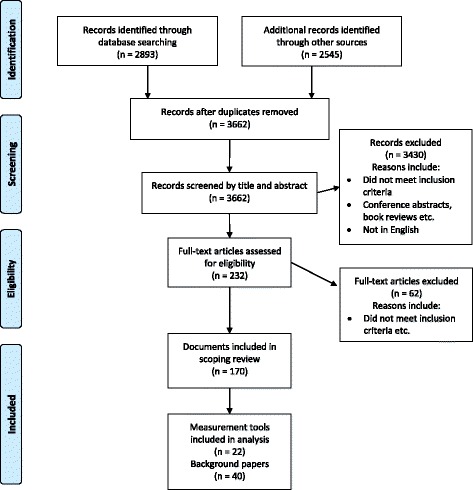
Contains the PRISMA flow diagram of the search process

The empiric and grey literature searches were carried out as detailed above. A total of 170 documents were included in the final scoping review. The process is displayed in the Preferred Reporting Items for Systematic Reviews and Meta-Analyses (PRISMA) flow diagram in Fig. 1 [39]. From these documents, 22 tools or measures for the assessment of individual-level social inclusion or social exclusion, or the measurement of very closely related concepts, were identified and charted in Table 1. Additional file 1 provides details on the background literature linked to each of the 22 tools described in Table 1. Having located the background literature naming each tool, attempts were made to contact the authors of each tool by email. They were asked to provide a copy of the original tool for scrutiny. One row of Table 1 is incomplete as we were unable to obtain the original Human Givens (HG) tool [40], and we had to rely on scant secondary information to describe this measure [41]. We have therefore used the denominator 21 (rather than 22 tools) when describing characteristics of the tools.

### Measurement tools discovered

Fourteen of the tools (14/21) mentioned in Table 1 below look at the measurement of the concept of social inclusion specifically, another tool (1/21) was described as a measure of both social inclusion and social exclusion, and then the remaining six tools discovered (6/21) did not mention the specific measurement of either concept in their supporting literature. Instead, they looked at the closely linked concepts of participation, integration, recovery and vulnerability. There were no scales or tools found that were developed for the measurement of social exclusion alone.

The majority (15/21) of the tools were developed and used with patients attending mental health services of some kind. A further four of the tools (4/21) were devised for use with both those attending mental health services and other groups; including members of the general population, people who are migrants and those with traumatic brain injury. Only three (3/21) of the measures found were not to have been developed specifically with a focus on mental health; these were for use with people who experience homelessness and people with disabilities.

When exploring the origin and background of each of the tools the following became apparent:

- Four of the tools (4/21) were based on other earlier measures that have also been included in the Table 1; the Social and Community Opportunities Profile Chinese version (SCOPE-C) was based on the earlier Social and Community Opportunities Profile (SCOPE), the Composite Measure of Social Inclusion (CMSI) was partially based on the Community Integration Questionnaire (CIQ), the Living in the Community Questionnaire (LCQ) was based on the Activity and Participation Questionnaire (APQ-6) and the Mental Health Recovery Star (MHRS) was based on the Homeless Outcomes Star (HOS).
- Three of the tools (3/21) were based on previous quantitative surveys or national censuses carried out in their country of origin; the Social Inclusion Questionnaire User Experience (SInQUE), the Social Inclusion Scale/Measure (SIS/SIM) and the APQ-6.
- Two (2/21) tools developed for use in homeless service settings were described as having been developed to meet the specific needs of people there in a ‘bottom-up’ process involving staff and service users.

When reviewing the administration of the tools, the number of items or questions included in each tool varied; with 121 questions asked in the long version of the SCOPE too, compared to four questions in the measure of Multidimensional Social Inclusion (MSI). Three (3/21) of the tools had both a long and short version of the tool developed; the SCOPE, the Australian Community Participation Questionnaire (ACPQ) and the Participation Scale (PS). Completion of the majority (20/21) of the tools involved self-reported questionnaires or interviews of patients by staff of researchers. One tool (1/21), the Staff Survey of Social Inclusion (SSSI), was carried out by staff at the mental health service without the relevant patient being involved in the assessment.

The outputs of the tools varied substantially:

- 13/21 reported some form of a score of social exclusion or social inclusion.
- 3/21 looked at the total number of hours spent on activities that may be related to social inclusion (CMSI, APQ-6, SSSI).
- 3/21 resulted in the development of a visual map of the social inclusion status of the person in question (HOS, MHRS, Inclusion Web [IW]).
- 2/21 resulted in qualitative answers only that were available for further analysis, or used to prompt discussion between the person being assessed and relevant service providers (EMILIA Project Questionnaire [EPQ], MSI).

### Specific domains of measurement tools

Each of the 22 measurement tolls has been included in Table 2 below, displaying the domains covered in their questions. The most common domain seen was Social Networks (referred to in some way in all 22 tools); which included all aspects of interaction with family members and friends, and feeling accepted by them. Community and Safety (17/22) included the ideas of feeling part of community and feeling safe in that setting. The domain of Leisure, Cultural & Religious (14/22) was concerned with regularly taking part in these types of activities. Usefulness and Potential (7/22) was concerned with a person feeling able to contribute positively to society and being able to fulfil personal potential. Rights and Freedoms (3/22) looked at freedom to express oneself and being aware of personal rights. The Other category was utilised for domains that arose only once or twice when analysing the tools; including themes as diverse as political engagement, hopefulness and offending.

**Table 2:** Domains Included in Measurement Tools

Domains	Social Networks	Community & Safety	Leisure, Cultural & Religious	Employment	Education & Training	Medical & Health	Housing	Volunteering & Charity	Financial	Usefulness & Potential	Domestic Functioning	Stigma	Independence, Control & Identity	Rights & Freedoms	Addiction	Self-Care	Other
Tools																	
ACPQ	x	x	x		x			x									political engagement
APQ-6	x	x		x	x			x									readiness to change
CIM / CIQ	x	x	x				x			x							
CMSI	x	x		x	x	x		x			x	x					
EPQ	x			x	x			x									
ESIQ	x	x	x	x	x		x			x		x		x			treatment by services
HG	x	x	x			x			x	x			x				give and receive attention, creativity
HOS	x					x	x		x	x	x				x	x	motivation and taking responsibility, offending
IW	x		x	x	x			x									
LCQ	x	x	x	x	x	x	x	x		x	x						happiness, hopefulness
MHRS	x	x		x		x					x		x		x		trust and hope, responsibilities
MSI	x	x	x	x		x	x		x	x		x	x			x	services, transport, belonging, quality of life, risk taking, recovery, coping, roles
PS	x	x	x		x											x	general tasks and demands, communication, mobility, major life areas
SCOPE	x	x	x	x	x	x	x		x								overall inclusion
SCOPE-C	x	x	x	x	x	x	x		x								
SInQUE	x		x	x	x	x	x	x	x								political engagement
SIQ	x	x				x											
SIS	x		x	x					x		x						
SIS / SIM	x	x	x				x	x		x		x		x			
SNQ	x	x			x	x			x		x		x				
SSSI	x	x	x	x	x			x									
VAT	x	x				x	x		x					x	x		survival skills, clothing and food, organisation, communication
Total = 22	22	17	14	13	13	12	10	9	9	7	6	4	4	3	3	3	

### Definitions

There are a wide variety of definitions of both social exclusion and social inclusion documented in the literature. Several review papers list some of the many definitions, and compare the elements that these definitions do and do not include [9, 14, 42, 43]. Additional file 1 summarises the background literature relating to each of the 22 tools selected for this scoping review. It is notable that many of the papers cited in Additional file 1 do not have a clear definition of what is meant by social exclusion or social inclusion, despite discussing the measurement of these concepts. Some authors did not definitively choose any one definition and instead listed a number of existing ones, while others combined elements of various definitions in an effort to provide clarity [41, 44, 45]. Of the papers that did set out a clear definition at the outset, it was one from the World Bank that appeared most often [46]. One paper included a definition of social inclusion apparently composed by the authors themselves [47]. A selection of the definitions cited in the papers is included in Table 1, Additional file 1 and other commonly cited definitions are listed in Tables 3 and 4 below.

**Table 3:** Definitions of Social Exclusion

Author(s)	Definition
Room 1997 [100]	Social exclusion focuses primarily on relational issues - inadequate social participation, lack of social integration and lack of power.
Brennan et al. 1998 [101]	Those people who [are socially excluded] do not have the means, material or otherwise, to participate in social, economic political and cultural life.
Power 2000 [102]	[Social exclusion is defined as] the inability of our society to keep all groups and individuals within reach of what we expect as a society and the tendency to push vulnerable people into the least popular places.
Sayce 2000 [60]	[Social exclusion involves] the interlocking and mutually compounding problems of impairment, discrimination, diminished social role, lack of economic and social participation and disability. Among the factors at play are lack of status, joblessness, lack of opportunities to establish family, small or non-existing social networks, compounding race and other discriminators, repeated rejection and consequent restrictions of hope and expectation.
Burchardt et al. 2002 [103]	Social exclusion occurs when an individual does not participate in key activities of the society in which he or she lives, for reasons beyond their control and in which they would like to ‘participate’.
Council for the European Union 2003 [61]	Social exclusion is a process whereby certain individuals are pushed to the edge of society and prevented from participating fully by virtue of their poverty, or lack of basic competencies and lifelong learning opportunities, or as a result of discrimination. This distances them from job, income and education opportunities as well as social and community networks and activities. They have little access to power and decision-making bodies and thus often feeling powerless and unable to take control over the decisions that affect their day to day lives.
UK Social Exclusion Unit 2004 [59]	Social exclusion is what can happen when people or areas suffer from a combination of linked problems such as unemployment, poor skills, low incomes, poor housing, high crime, poor health and family breakdown. In the past, governments tried to deal with each of the problems of social exclusion individually, but there was little success in tackling the complicated links between them, or preventing problems from arising in the first place.
Levitas et al. 2007 [104]	Social exclusion is a complex and multi-dimensional process. It involves the lack or denial of resources, rights, goods and services, and the inability to participate in the normal relationships and activities, available to the majority of people in a society, whether in economic, social, cultural or political arenas. It affects both the quality of life of individuals and the equity and cohesion of society as a whole.
Popay et al. 2008 (WHO SEKN Report) [8]	Exclusion consists of dynamic, multi-dimensional processes driven by unequal power relationships. These operate along and interact across four dimensions - cultural, economic, political and social and at different levels including individuals, groups, households, communities, countries and global regions. Exclusionary processes contribute to health inequalities by creating a continuum of inclusion/exclusion. This continuum is characterised by an unjust distribution of resources and unequal capabilities and rights required to: create the conditions necessary for entire populations to meet and exceed basic needs, enable participatory and cohesive social systems, value diversity, guarantee peace and human rights, sustain environmental systems.

**Table 4:** Definitions of Social Inclusion

Author(s)	Definition
Sayce 2001 [105]	[Social inclusion is] a virtuous circle of improved rights of access to the social and economic world, new opportunities, recovery of status and meaning, and reduced impact of disability. Key issues will be availability of a range of opportunities that users can choose to pursue, with support and adjustment where necessary.
Bates and Repper 2001 [106]	[Social inclusion requires] full access to mainstream statutory and post sixteen education, open employment, and leisure opportunities alongside citizens who do not bear these [mental health] labels.
Council for the European Union 2003 [61]	Social inclusion is a process which ensures that those at risk of poverty and social exclusion gain the opportunities and resources necessary to participate fully in economic, social and cultural life and to enjoy a standard of living and well-being that is considered normal in the society in which they live. It ensures that they have greater participation in decision making which affects their lives and access to their fundamental rights (as defined in the Charter of Fundamental Rights of the EU).
Marino-Francis and Worrall-Davies [47]	Social inclusion is about each person taking part in society and having control over their own resources. It is also about a community that cares for its members, makes them feel welcome and is willing to adjust to fit their various needs.
World Bank 2013 [46]	[Social inclusion refers to] promoting equal access to opportunities, enabling everyone to contribute to social and economic programs and share in its rewards.
Killaspy et al. 2014 [88]	Social inclusion refers to the opportunities that individuals have to participate in key areas of economic, social and cultural life.

## Discussion

### Statement of principal findings

This scoping review found that the concepts of social inclusion and social exclusion, while often described as abstract and lacking clarity, have both been discussed and measured at the individual level in relation to health. This review identified 22 relevant measurement tools across the peer-reviewed and grey literature. The majority of these tools were developed for measuring these concepts in mental health settings, and it is not clear why this field predominates. It is also unclear as to why there are so many of these measurement tools, even in relation to mental health. The tools that are listed have been developed and utilised in a number of different countries, and by researchers from various backgrounds and disciplines.

### Discussion of findings

#### Tools

The number of tools that have been created since the year 2000 is striking. It is likely that the lack of agreement on definitions and the domains that should be included for measurement are factors. The background papers reported in Additional file 1 highlight that the work associated with these tools has been published in a wide variety of research areas including journals relating to psychiatry, general mental health, occupational therapy, disability, rehabilitation, development, homelessness and social inclusion itself. This highlights the point that the concepts of social inclusion and social exclusion are felt to be relevant to researchers and practitioners across many disciplines, but this may have led to duplication when it came to the development of measurement tools.

It is obvious that the concepts of social inclusion and exclusion are of great importance to mental health researchers and clinicians. Authors have explained that the social exclusion can contribute to mental illness, but also “improving social inclusion of the individual is an important contributor to recovery” [48, 49]. The measurement of social inclusion status and its changes over time in patients who are engaging with treatment for mental health problems are seen as tangible outcomes in mental health clinical settings; they are considered useful alongside the more traditional measures of symptom control. One report on mental health promotion explains that “social inclusion for an individual means access to supportive relationships, involvement in group activities and civic engagement” [50]. Encouraging the social inclusion and reintegration of people with mental health problems into society has also become an important policy goal internationally [51, 52]. One possible reason for this is recognition of the immense, and increasing, economic and social burden of mental ill health worldwide [53–55]. The basis for this goal is the idea that an individual with mental illness who receives appropriate and timely treatment will eventually become more engaged and included in society, making it more likely that they will be able to re-enter the workforce and contribute.

The literature pertaining to two of the tools in particular, the SCOPE and the LCQ, highlighted that the authors had conducted extensive searches for existing measures of social exclusion and social inclusion prior to beginning their own work. Both tools were developed as part of large-scale commissioned research projects: with the SCOPE tool resulting from work on a Health Technology Assessment carried out for the UK’s National Institute for Health Research, and the LCQ being developed by the Australian Mental Health Outcomes and Classification Network for their Government Mental Health Information Strategy Standing Committee. The creators of four other tools (SCOPE-C, CMSI, LCQ and MHRS) also explained that their particular measure was based on an existing tool. For example, the SCOPE-C was developed by adapting the SCOPE tool from the UK for the language and cultural context of people in Hong Kong. The researchers conducted qualitative studies on the meaning of social inclusion in that country and then altered the domains and questions asked as part of the tool accordingly. The LCQ was a tool produced by adding questions on topics such as housing and physical health to the existing APQ-6 tool following feedback from relevant groups.

It is notable that none of the tools stated that its aim was to measure only social exclusion. Fourteen of the tools described their aim was to measure social inclusion, and one (the EPQ) indicated it was meant for the measurement of both social inclusion and exclusion. It is unclear why social exclusion is a less frequently used term in this context: it may be related to variations in the language used around the concepts of social inclusion and social exclusion, or the perception that social exclusion is more difficult to measure when compared with social inclusion. Many authors use both terms when explaining the one issue, for example; “Despite efforts in Europe to enhance social inclusion of mental health service users, they still remain a highly socially excluded group” [56]. This implies that inclusion and exclusion are the opposites of each other. This may then lead to the presumption that if you measure social inclusion, you have assessed both social inclusion and social exclusion status.

There are a number of concepts very closely aligned with social inclusion and exclusion that were measured by seven of the 22 tools described. For example, the SIS tool for patients in mental health settings with schizophrenia focuses on the concept of social integration. When we look more closely at this tool, all of the domains it covers overlap with those of other social inclusion measures described in Table 2. The authors of a review on social inclusion and global mental health that included this SIS tool explained that “a variety of terms, including ‘social inclusion’ and ‘social integration’, are used interchangeably in both research and policy documents” [57]. While the definitions of social inclusion and social exclusion themselves are unclear, the fact that authors and policy makers also use other similar, but equally ill-defined, terms in discussion of these complex concepts may add to the confusion around the issue. Other tools included in Table 1 seek to assess concepts such as participation, recovery and vulnerability. The domains these tools cover are also very similar to the domains covered by those tools explicitly stating that they are measures of social inclusion.

The SEKN report was critical of the approach taken by many researchers and policy makers who had discussed social exclusion as a state, rather than focussing on the exclusionary processes that led to and perpetuated that vulnerable state [8]. The work of the SEKN could have offered some clarity on questions relating to the concept of social exclusion, and yet there is little mention of the report in the background literature of the tools that were published after 2008. Subsequent research on social exclusion measurement did not seem to rely on the SEKN report for reference or a definition of the concept of social exclusion. This may have been because the SEKN team only discussed the measurement of exclusion at global, regional and country level; there was no analysis of individual level measurement. More recently, some authors such as Adam and Potvin have taken the work of the SEKN and adapted it to focus more on individual level social exclusion [58]. Another reason the SEKN work may not feature is that the SEKN authors stated “inclusion in some measures of a variety of health indicators as a component of or risk factor for social exclusion, rather than an outcome of the experience, all make it difficult to ‘measure’ the impact of social exclusion in health outcomes”. This statement highlighted possible confusion around the many factors that may lead to social exclusion, compared with those that may have resulted from it. Finally, the SEKN authors reported that social exclusion was too complex a subject to be adequately assessed by quantitative methods alone, declaring that “exclusionary processes can only be adequately ‘represented’ through both quantitative and qualitative data – through both indicators and stories” [8]. The majority of the individual level tools detailed in Table 1 result in quantitative scores or some other measure of social inclusion or social exclusion, and only two tools collected qualitative answers. This may have been because quantitative scores are easier to conduct and to repeat over time in busy clinical settings, and this is precisely where tools included were mainly intended for use.

Significantly, none of the 22 individual tools discovered were specifically developed or used in general primary healthcare settings. As primary healthcare is the point of initial contact the majority of people have with the health system and it includes such a wide variety of components, it can offer help for many health issues. Primary healthcare, and universal health coverage in particular, is discussed as part of the solution to many of the causes and end results of social exclusion. It would therefore seem to be a logical place to try to assess and monitor social inclusion and social exclusion – but this does not yet appear to be the case.

#### Domains

It makes sense that the two domains most frequently found in the 22 individual tools displayed in Table 2 are Social Networks and Community; these are referred to in 22/22 and 17/22 respectively. What is surprising, however, is that many tools omit domains that would seem to be important for any measure of social inclusion or social exclusion to cover. For example, ‘housing’ is only mentioned in 10/22. Having somewhere secure to live tends to be considered a fundamental need to be dealt with before more complex issues such as health problems can be addressed. References to important issues such as Stigma and knowledge of Rights and Freedoms are even less frequently seen in the tools included, being included in only 4/22 and 3/22 respectively. In addition, the fact that there are over thirty different domains mentioned across the 22 tools we investigated highlights the fact that work in this area is hampered by the lack of a consensus definition and agreement on the domains that should be accounted for in any measure.

#### Definitions

As stated, there are multiple definitions of both social exclusion and social inclusion across the published and grey literature. Several reports and papers tabulate and compare the various definitions [11, 14, 43]. Looking at the selection of definitions of social exclusion in Table 3, it is notable that only those of the UK SEU [59] and the WHO SEKN [8] specifically mentioned health as a factor to be considered in relation to exclusion. The language used by the creators of the various definitions is very interesting; particularly around those of social inclusion (Table 4), with authors using very positive and encouraging words and phrases such as “virtuous circle of improved rights”, “new opportunities”, “full access”, “having control”, “a community that cares” and “enabling everyone”. These terms could be considered more optimistic and acceptable than the negative terms often used to explain the concept of social exclusion. This may explain why governments and others have adopted the positive language of social inclusion when developing policies or even establishing initiatives (e.g. the European Social Inclusion Strategy, the Australian Social Inclusion Board).

Definitions of social exclusion can be broadly categorised: some address the problems associated with exclusion [59, 60], others detail what aspects of life people are excluded from [61], and others mention the various levels on which exclusion is seen to operate [8]. Room described many of the recurring ideas seen across various definitions when he stated that social exclusion was ultimately a “multidimensional, dynamic and relational concept” [14, 62, 63].

### Strengths and weaknesses of this study

The strengths of this scoping review include the fact that a wide range of databases and grey literature sources were searched by the authors. Manual searches of the reference lists of included publications were carried out, and we attempted to contact all relevant tool authors. This resulting review also contains publications across a number of disciplines, and work from a variety of countries is included. We included a number of review papers, mostly looking at mental health, adding to the likelihood that all relevant individual measurement tools were included.

There were a number of limitations to this scoping review. Firstly, critical appraisal of the background papers or the resulting tools was not included as this was beyond the scope of this type of review. Some of the 22 tools included were previously validated and evaluated, others were not, and this was not taken into account for this publication. The authors were unable to contact all tool authors; this meant having to rely on secondary sources for descriptions of some tools, leaving some sections of the tables incomplete. The searches carried out were limited to papers in English, and those published since the year 2000.

## Conclusions

This scoping review offers a comprehensive description of existing work on the measurement at the individual level in healthcare settings of social exclusion and social inclusion. We have firstly shown that there is a wide range of definitions of both terms in use, and they tend to focus on quite different aspects of social exclusion and social inclusion. Some definitions describe the problems associated with social exclusion, others mention the parts of life that people are excluded from, and others explain the levels that social exclusion operates on. We have listed the measurement tools developed for use with individual patients in healthcare settings. These tools vary in the number of items they include, how scores are allocated and in how they are administered. The majority of these tools were designed for use in mental health settings. These tools cover a wide variety of domains, perhaps highlighting the differing views of researchers and practitioners on what exactly is meant by the terms social exclusion and social inclusion. There is apparently no measurement tool intended specifically for use in primary healthcare settings for the measurement and monitoring of changes in social inclusion or social exclusion status. It would appear, therefore, that there is scope to develop a measurement tool for this purpose, or to modify an existing tool that covers most or all of the domains felt to be important in the context of primary healthcare.

## Additional file

Additional file 1: Contains the background literature for each measurement tool in Tables 1 and 2. (DOCX 26 kb)

## Abbreviations

ACPQ: Australian community participation questionnaire
APQ-6: Activity and participation questionnaire
CIM / CIQ: Community integration measure / questionnaire
CMSI: Composite measure of social inclusion
DESC: Downtown emergency service centre
EMILIA: Empowerment of mental illness service users: lifelong learning and action
EPQ: EMILIA project questionnaire
ESIQ: Evaluating social inclusion questionnaire
GP: General practitioner
HG: Based on ‘The Human Givens’
HK: Hong Kong
HOS: Homeless outcomes star
IW: Inclusion web
LCQ: Living in the community questionnaire
MHRS: Mental health recovery star
MSI: Multidimensional social inclusion
NHS: National Health Service
PRISMA: Preferred reporting items for systematic reviews and meta-analyses
PS: Participation scale
RCGP: Royal College of general practitioners
SCOPE: Social and community opportunities profile
SCOPE-C: Social and community opportunities profile – Chinese version
SDG: Sustainable development goal
SE: Social exclusion
SEKN: Social exclusion knowledge network
SEU: Social exclusion unit
SI: Social inclusion
SInQUE: Social inclusion questionnaire user experience
SIQ: Social inclusion questionnaire
SIS / SIM: Social inclusion scale / Social inclusion measure
SIS: Social integration survey
SNQ: Support needs questionnaire
SSSI: Staff survey of social inclusion
UK: United Kingdom
USA: United States of America
VAT: Vulnerability assessment tool
WHO: World Health Organization
