# Supplementary material for: Measuring social exclusion in healthcare settings: a scoping review
Source: Int J Equity Health. 2018 Feb 2;17:15. doi: 10.1186/s12939-018-0732-1 (PMC5796599; doi:10.1186/s12939-018-0732-1)
Supplement: Additional file 1: — Contains the background literature for each measurement tool in Tables 1 and 2. (DOCX 26 kb) [file 12939_2018_732_MOESM1_ESM.docx]

Additional File 1: Background literature for each Tool

| **Name** | **Papers Linked** | **Country** | **Publication Type** | **Definition Of Social Exclusion or Inclusion** | **Purpose Of This Paper** | **Study Characteristics** | **Review Paper** |
| --- | --- | --- | --- | --- | --- | --- | --- |
| SInQUE | Mezey et al. 2012 [1] | United Kingdom (UK) | peer reviewed, International Journal of Social Psychiatry | lists multiple incl Burchardt et al. 2002 [2], Huxley and Thornicroft, 2003 [3], Morgan et al. 2007 [4] | develop and test validity of measure of social inclusion (SI) for individuals with severe mental illness | 66 participants, structured interview | Baumgartner and Burns 2014 [5] |
|  | Killaspy et al. 2014 [6] | UK | peer reviewed, International Journal of Social Psychiatry | Burchardt et al. 1999 [7] | investigate changes in SI after development of psychotic Illness and associated factors | 67 participants, cross-sectional survey that collected current and retrospective data from participants to allow for comparisons of SI before and after the development of psychotic illness | Baumgartner and Burns 2014 [5] |
| SCOPE | Huxley et al. 2006 [8] | UK | report for National Coordinating Centre for Research Methodology | combines multiple incl Brennan et al. 1998 [9] , Room 1997 [10], Berman and Phillips 2000 [11] | review existing SI measures and literature, describe SI concept mapping exercises conducted with different groups incl mental health service users, professionals and members of the general population | 66 participants in 9 groups |  |
|  | Huxley et al. 2012 [12] | UK | report on Health Technology Assessment for National Institute for Health Research | lists multiple incl Brennan et al 1999, Room, 1997, Berman and Phillips 2000 | psychometric development of SI index to capture subjective and objective life domains, development of a long and short version | tested in general community, on students and patients mental health settings | Baumgartner and Burns 2014 [5], Coombs et al. 2013 [13], AMHOCN 2015 [14] |
| SCOPE-C | Chan et al. 2014 [15] | Hong Kong (HK) | peer reviewed, Social Indicator Research | lists multiple incl Huxley et al. 2006 [8], Burchardt et al. 2002 [2, 16], European Commission 2004 [17] | focus group study on meaning of SI involving concept mapping, to inform development of Chinese-language measure of SI (SCOPE-C) | 7 groups involving 61 participants including NGO staff, senior centre users, community residents, people with severe mental illness, professional social service providers, communication studies & social work students |  |
|  | Chan et al. 2015 [18] | HK | peer reviewed, Social Indicator Research | World Bank 2013 [19] | demographics recorded, then measure experience of SI of mental health patients using SCOPE-C and two other measures | 168 participants, face-to-face individual interviews |  |
|  | Huxley et al. 2016 [20] | UK & HK | peer reviewed, International Journal of Social Psychiatry | Council for the European Union 2003 [21] | report on analysis of structural equivalence and item differentiation, comparing two country results | 416 participants, mental health patients and general population in HK & UK |  |
|  | Chan et al. 2016 [22] | UK & HK | peer reviewed, Social Indicator Research | World Bank 2013 [19] | report on SI and correlation with health conditions among Chinese immigrants in HK & UK | 56 participants in HK, 51 in UK |  |
|  | Chan et al. 2016 [23] | Hong Kong | peer reviewed, Community Mental Health Journal | World Bank 2013 [24] | to describes construction of Chinese version of SCOPE (SCOPE-C) and to measure SI among mental health services users in HK | 168 participants, questionnaire with 56 items |  |
| SIS / SIM | Secker et al. 2009 [25] | UK | peer reviewed, Journal of Mental Health | none of SI/SE | to develop SI measure for use in study assessing outcomes of arts participation for people with mental health needs | 23 arts participants/service users and 88 arts project participants, questionnaires | Baumgartner and Burns 2014 [5], Coombs et al. 2013 [13], AMHOCN 2015 [14], Wright and Stickley 2013 [26] |
|  | Wilson and Secker 2015 [27] | UK | peer reviewed, Social Inclusion | Commission of the European Communities 2000 [28] | to validate the SIS in sample of university students, reliability, consistency and validity were assessed by comparing SIS scores with scores on other measures of SI | 103 participants first round, then 95 of these again in second round, questionnaires |  |
|  | Margrove et al. 2013 [29] | UK | unclear, Perspectives in Public Health | none of SI/SE | to conduct waiting list controlled programme evaluation of course in arts and mental health | 32 in control group, 26 in intervention group, questionnaire and focus groups | Baumgartner and Burns 2014 [5] |
| SIS | Kawata and Revicki 2008 [30] | United States of Americs (USA) | peer reviewed, Quality of Life Research | none of SI/SE | use SIS tool to measure social functioning in schizophrenia from patient and informant perspective, comparing SIS to other measures | 129 patients, has patient self-reported part and observer part | Baumgartner and Burns 2014 [5] |
| CIM / CIQ | McColl et al. 2001 [31] | Canada | peer reviewed, Archives of Physical Medicine and Rehabilitation | none of SI/SE | to describe development of CIM and its psychometric properties | 92 participants; 41 had acquired brain injuries, 36 college students and 15 family members | Baumgartner and Burns 2014 [5] |
|  | Lloyd et al. 2010 [32] | Australia | peer reviewed, International Journal of Social Psychiatry | none of SI/SE | to determine whether subjective dimensions of recovery such as empowerment are associated with self-report of more objective indicators such as participation in community and income | 161 participants with severe mental illness | Baumgartner and Burns 2014 [5] |
| CMSI | Lloyd et al. 2008 [33] | Australia | peer reviewed, Australian Occupational Therapy Journal | none | to assess reliability of components of proposed CMSI for people with psychiatric disabilities | 54 participants, face-to-face interviews | Coombs et al. 2013 [13], AMHOCN 2015 [14], Wright and Stickley 2013 [26] |
| APQ-6 | Stewart et al. 2010 [34] | Australia | peer reviewed, Australian and New Zealand Journal of Psychiatry | none of SI/SE | to report on development and reliability of APQ6, focus in Participation and Recovery | 129 mental health service users, self-report measure | Coombs et al. 2013 [13], AMHOCN 2015 [14] |
| ACPQ | Berry et al. 2007 [35] | Australia | peer reviewed, Social Science & Medicine | none of SI/SE | to report on development of questionnaire and its validation with a focus on community participation | 963 randomly selected community members, anonymous postal survey | Coombs et al. 2013 [13], AMHOCN 2015 [14] |
| EPQ | Ramon et al. 2009 [36] | eight European sites | peer reviewed, International Journal of Social Psychiatry | none explicit, but lists Griffiths 2006 [37] and Davidson et al. 2001 [38] | to examine changes in SE/SI of mental health service users who took part in programme [Empowerment of Mental Illness Service Users: Lifelong Learning and Action (EMILIA)] across eight European sites | semi structured interviews and self-reports of service users, before and after case study design - baseline and 10 months later | Coombs et al. 2013 [13], AMHOCN 2015 [14] |
| ESIQ | Stickley and Shaw 2006 [39] | UK | non peer reviewed, mental health practice | Sayce 2000 [40] | to report on development and piloting of questionnaire on SI | semi structured interview | Coombs et al. 2013 [13] |
| IW | Hacking and Bates 2008 [41] | UK | peer reviewed, Mental Health Review Journal | none explicit, but lists Sayce 2001 [42], Hacking 2005 [43], Spandler 2007 [44] | to describe pilot study and explain the Inclusion Web tool | discussion with prompts and map generation, repeated over time allowing monitoring | Coombs et al. 2013 [13], AMHOCN 2015 [14] |
| SIQ | Marino-Francis and Worrall-Davies 2010 [45] | UK | peer reviewed, Mental Health Review Journal | authors own definition | to report on development and validation of tool for measuring SI of mental health day service users | 69 participants, self-reported, Likert scale | Coombs et al. 2013 [13], AMHOCN 2015 [14] |
| SSSI | Dorer et al. 2009 [46] | UK | peer reviewed, British Journal of Occupational Therapy | Bates and Repper 2001 [47] | to report use of the tool on people with long-term mental health problems in inner-city mental health rehabilitation service | 199 service users, staff report estimate of time spent on activities by service user over one week | Coombs et al. 2013 [13], AMHOCN 2015 [14] |
| LCQ | Coombs et al. 2016 [48] | Australia | peer reviewed, Asia Pacific Journal of Social Work and Development | none explicit, but describes SI indicators from Australian Health Ministers 2009 [49] | to report development and initial psychometric testing, both objective and subjective elements included | 244 mental health service users in pilot, 1001 of general public, self-report |  |
|  | AMHOCN 2015 [14] | Australia | report for Government Mental Health Information Strategy Standing Committee | lists various definitions including Sayce 2001 [42], Bates and Repper 2001 [47], Marino-Francis and Worrall-Davies 2010 [45] | to review mental health & SI literature and individual-level measures, to report consultations with relevant people, to report psychometric testing of resulting tool |  |  |
| HG | Williamson and Allen 2006 [50] in Huxley et al. 2006 [8] |  |  |  |  |  |  |
| SNQ | Davis and Burns 2015 [51] | UK | peer reviewed, Social Inclusion | none of SI/SE | to describe design and development of tool, and report on its reliability and validity | 7 staff on 82 service users, tool completed by staff |  |
| HOS | Johnson and Pleace 2016 [52] | international | peer reviewed, European Journal of Homelessness | none of SI/SE | to examine ideological framework, as well as theoretical and methodological approaches | completed by service user and key worker together, visual mapping of situation |  |
|  | Burns et al. 2008 [53] | UK | commissioned report | none of SI/SE | to report impact of using the tool on service delivery and lessons learned about effective implementation | interviewed managers in 25 organisations that had been using the tool |  |
|  | Peteresen et al. 2014 [54] | USA | peer reviewed, Clinical Scholars Review | none of SI/SE | to report on the use of HOS in one homeless shelter | 10 homeless participants |  |
| MHRS | Onifade 2011 [55] | UK | peer reviewed, Mental Health and Social Inclusion | none of SI/SE | to describe origin and development of tool | completed by service user and key worker together, visual mapping of situation |  |
|  | Dickens et al. 2012 [56] | UK | peer reviewed, The Psychiatrist (now BJPsych Bulletin) | none of SI/SE | to explore its factorial validity, internal consistency and responsiveness | readings conducted twice with 203 adults with moderate to severe mental health problems |  |
|  | Killaspy et al. 2012 [57] | UK | peer reviewed, British Journal of Psychiatry | none of SI/SE | to assess psychometric properties of MHRS | 172 services users and 120 staff from in-patient and community services participated, ratings agreed through discussion between service user and mental health worker lasting approx. 1hr |  |
| VAT | Downtown Emergency Service Centre (DESC) 2009 [58] | USA | online report | none of SI/SE | to describe the background and development of VAT tool | designed for use by service providers interviewing homeless people |  |
|  | Ginzler and Monroe-DeVita 2010 [59] | USA | commissioned report authored by Washington Institute for Mental Health Research and Training at University of Washington | none of SI/SE | to conduct a psychometric assessment of the VAT instrument | 277 interviews and 171 follow-up interviews with new or continuing clients |  |
| PS | van Brakel et al. 2006 [60] | international | peer reviewed, Disability and Rehabilitation | none of SI/SE | to develop and validate a measure of social participation | participants rate own participation in comparison with a peer (who does not have disability), Nepal, India and Brazil |  |
| MSI | le Boutillier and Croucher 2010 [61] | UK | peer reviewed opinion piece, British Journal of Occupational Therapy | Sayce 2001 [42] | to describe practical tool for examining SI |  |  |

References:

1. Mezey G, White S, Thachil A, Berg R, Kallumparam S, Nasiruddin O et al. Development and preliminary validation of a measure of social inclusion for use in people with mental health problems: The SInQUE. International Journal of Social Psychiatry. 2012;59(5):501-7.
2. Burchardt T, Le Grand J, Piachaud D. Degrees of Exclusion: Developing a Dynamic, Multidimensional Measure. In: Hills J, Le Grand J, Piachaud D, editors. Understanding Social Exclusion. Oxford: Oxford University Press; 2002. p. 30-43.
3. Huxley P, Thornicroft G. Social inclusion, social quality and mental illness. British Journal of Psychiatry. 2003;182(4):289-90.
4. Morgan C, Burns T, Fitzpatrick R, Pinfold V, Priebe S. Social exclusion and mental health: conceptual and methodological review. British Journal Of Psychiatry. 2007;191:477-83.
5. Baumgartner JN, Burns JK. Measuring social inclusion—a key outcome in global mental health. International Journal of Epidemiology. 2014;43(2):354-64.
6. Killaspy H, White S, Lalvani N, Berg R, Thachil A, Kallumpuram S et al. The impact of psychosis on social inclusion and associated factors. International Journal of Social Psychiatry. 2014;60(2):148-54.
7. Burchardt T, Le Grand J, Piachaud D. Social exclusion in Britain 1991—1995. Social Policy & Administration. 1999;33(3):227-44.
8. Huxley P, Evans S, Munroe M, Webber M, Burchardt T, Knapp M et al. Development of a Social Inclusion Index to capture subjective and objective domains (Phase I): National Co-ordinating Centre for Research and Methodology. 2006. http://citeseerx.ist.psu.edu/viewdoc/download?doi=10.1.1.469.5761&rep=rep1&type=pdf. Accessed 14 Jul 2016.
9. Brennan A, Rhodes J, Tyler P. New findings on the nature of economic and social exclusion in England and the implications for new policy initiatives: Discussion paper 101. Cambridge: Department of Land Economy, University of Cambridge; 1998.
10. Room G. Social Quality of Europe: Perspectives on Social Exclusion. In: Beck W, Van der Maesen L, editors. The Social Quality of Europe. The Hague: Kluwer Law International; 1997. p. 255-62.
11. Berman Y, Phillips D. Indicators of social quality and social exclusion at national and community level. Social Indicators Research. 2000;50(3):329-50.
12. Huxley P, Evans S, Madge S, Webber M, Burchardt T, McDaid D et al. Development of a social inclusion index to capture subjective and objective life domains (Phase II): Psychometric development study. Health Technology Assessment. 2012. doi:10.3310/hta16010
13. Coombs T, Nicholas A, Pirkis J. A review of social inclusion measures. Australian & New Zealand Journal of Psychiatry. 2013;47(10):906-19.
14. Australian Mental Health Outcomes and Classification Network (AMHOCN). Development of the Living in the Community (LCQ) measure of social inclusion for use in mental health Final report: Australian Mental Health Outcomes and Classification Network (AMHOCN). 2015. http://www.amhocn.org/sites/default/files/publication_files/living_in_the_community_questionnaire_lcq_final_report.pdf. Accessed 01/10/2016.
15. Chan K, Evans S, Ng Y-L, Chiu MY-L, Huxley PJ. A concept mapping study on social inclusion in Hong Kong. Social Indicators Research. 2014;119(1):121-37.
16. Burchardt T, Le Grand J, Piachaud D. Introduction. In: Hills J, Le Grand J, Piachaud D, editors. Understanding Social Exclusion. Oxford: Oxford University Press; 2002. p. 1-12.
17. European Commission. Joint Report on Social Inclusion: Employment and Social Affairs.: Directorate-General for Employment and Social Affairs. 2004. http://ec.europa.eu/employment_social/social_inclusion/docs/final_joint_inclusion_report_2003_en.pdf. Accessed 28 Jun 2017.
18. Chan K, Evans S, Chiu MY-L, Huxley PJ, Ng Y-L. Relationship between health, experience of discrimination, and social inclusion among mental health service users in Hong Kong. Social Indicators Research. 2015;124(1):127-39.
19. The World Bank. Social development: Sector results profile. The World Bank. 2013. http://www.worldbank.org/en/results/2013/04/14/social-development-results-profile. Accessed 24 Jul 2017.
20. Huxley PJ, Chan K, Chiu M, Ma Y, Gaze S, Evans S. The social and community opportunities profile social inclusion measure: Structural equivalence and differential item functioning in community mental health residents in Hong Kong and the United Kingdom. International Journal of Social Psychiatry. 2016;62(2):133-40.
21. Council for the European Union. Joint Report by the Commission and the Council on Social Inclusion: Council for the European Union. 2003. http://ec.europa.eu/employment_social/soc-prot/soc-incl/final_joint_inclusion_report_2003_en.pdf. Accessed 12 Jul 2017.
22. Chan K, Huxley PJ, Chiu MY-L, Evans S, Ma Y. Social inclusion and health conditions among Chinese immigrants in Hong Kong and the United Kingdom: An exploratory study. Social Indicators Research. 2016;126(2):657-72.
23. Chan K, Chiu MY-L, Evans S, Huxley PJ, Ng Y-L. Application of SCOPE-C to measure social inclusion among mental health services users in Hong Kong. Community Mental Health Journal. 2016;52(8):1113-7.
24. The World Bank. Social Inclusion. The World Bank. 2013. http://www.worldbank.org/en/topic/socialdevelopment/brief/social-inclusion. Accessed 20 Jul 2017.
25. Secker J, Hacking S, Kent L, Shenton J, Spandler H. Development of a measure of social inclusion for arts and mental health project participants. Journal of Mental Health. 2009;18(1):65-72.
26. Wright N, Stickley T. Concepts of social inclusion, exclusion and mental health: a review of the international literature. Journal of Psychiatric and Mental Health Nursing. 2013;20(1):71-81.
27. Wilson C, Secker J. Validation of the Social Inclusion Scale with Students. Social Inclusion. 2015;3(4):52-62.
28. Commission of the European Communities. Social Policy Agenda: Communication from the Commission to the Council, the European Parliament, the Economic and Social Committee and the Committee of the Regions. Brussels: Commission of the European Communities. 2000. http://casentino.toscana.it/agenda21/a21/news/Risultati/doc/social-policy-agenda.pdf. Accessed 14 May 2017.
29. Margrove KL, Heydinrych K, Secker J. Waiting list-controlled evaluation of a participatory arts course for people experiencing mental health problems. Perspectives in Public Health. 2013;133(1):28-35.
30. Kawata AK, Revicki DA. Reliability and validity of the social integration survey (SIS) in patients with schizophrenia. Quality of Life Research. 2008;17(1):123-35.
31. McColl MA, Davies D, Carlson P, Johnston J, Minnes P. The community integration measure: development and preliminary validation. Archives of physical medicine and rehabilitation. 2001;82(4):429-34.
32. Lloyd C, King R, Moore L. Subjective and objective indicators of recovery in severe mental illness: a cross-sectional study. International Journal of Social Psychiatry. 2010;56(3):220-9.
33. Lloyd C, Waghorn G, Best M, Gemmell S. Reliability of a composite measure of social inclusion for people with psychiatric disabilities. Australian Occupational Therapy Journal. 2008;55(1):47-56.
34. Stewart G, Sara G, Harris M, Waghorn G, Hall A, Sivarajasingam S et al. A brief measure of vocational activity and community participation: development and reliability of the Activity and Participation Questionnaire. Australian & New Zealand Journal of Psychiatry. 2010;44(3):258-66.
35. Berry HL, Rodgers B, Dear KB. Preliminary development and validation of an Australian community participation questionnaire: Types of participation and associations with distress in a coastal community. Social Science & Medicine. 2007;64(8):1719-37.
36. Ramon S, Griffiths CA, Nieminen I, Pedersen M, Dawson I. Towards social inclusion through lifelong learning in mental health: analysis of change in the lives of the EMILIA project service users. International Journal of Social Psychiatry. 2009;57(3):211-23.
37. Griffiths CA. The theories, mechanisms, benefits, and practical delivery of psychosocial educational interventions for people with mental health disorders. International Journal of Psychosocial Rehabilitation. 2006;11(1).
38. Davidson L, Stayner DA, Nickou C, Styron TH, Rowe M, Chinman ML. "Simply to be let in": inclusion as a basis for recovery. Psychiatric Rehabilitation Journal. 2001;24(4):375-88.
39. Stickley T, Shaw R. Evaluating social inclusion. Mental Health Practice. 2006;9(10):14-21.
40. Sayce L. From Psychiatric Patient to Citizen: Overcoming Discrimination and Social Exclusion. London: Palgrave MacMillan; 2000.
41. Hacking S, Bates P. The inclusion web: a tool for person-centered planning and service evaluation. Mental Health Review Journal. 2008;13(2):4-15.
42. Sayce L. Social inclusion and mental health. Psychiatric Bulletin. 2001;25(4):121-3.
43. Hacking S. Social quality in Britain: a welfare state? European Journal of Social Quality. 2005;5(1):43-66.
44. Spandler H. From social exclusion to inclusion? A critique of the inclusion imperative in mental health. Medical Sociology Online. 2007;2(2):3-16.
45. Marino-Francis F, Worrall-Davies A. Development and validation of a social inclusion questionnaire to evaluate the impact of attending a modernised mental health day service. Mental Health Review Journal. 2010;15(1):37-48.
46. Dorer G, Harries P, Marston L. Measuring social inclusion: A staff survey of mental health service users' participation in community occupations. British Journal of Occupational Therapy. 2009;72(12):520-30.
47. Bates P, Repper J. Social inclusion ‐ A framework for evaluation. A Life in the Day. 2001;5(2):18-23.
48. Coombs T, Reed C, Rosen A. Developing the living in the community questionnaire: reporting the social outcomes of mental health care. Asia Pacific Journal of Social Work and Development. 2016;26(2-3):178-89.
49. Australian Health Ministers. Fourth National Mental Health Plan: An agenda for collaborative government action in mental health 2009-2014: Australian Health Ministers. 2009. https://www.health.gov.au/internet/main/publishing.nsf/content/9A5A0E8BDFC55D3BCA257BF0001C1B1C/$File/plan09v2.pdf. Accessed 12 Mar 2017.
50. Williamson M, Allen A. The Human Givens. Exeter: Mind South West. 2006.
51. Davis FA, Burns J. The Development and Properties of the Support Needs Questionnaire. Social Inclusion. 2015;3(4):63-75.
52. Johnson G, Pleace N. How Do We Measure Success in Homelessness Services?: Critically Assessing the Rise of the Homelessness Outcomes Star. European Journal of Homelessness. 2016;10(1):31-51.
53. Burns S, MacKeith J, Graham K. Using the Outcomes Star. Impact and Good Practice: Triangle Consulting. 2008. http://anichaconsulting.com.au/pdf/Outcomes-Star-Impact-and-good-practice.pdf. Accessed 14 Oct 2016.
54. Petersen VA, Ellis P, Lorenz R, Armbrecht E. An Interventional Study of Guiding Homeless Persons to Self-Reliance Using the Outcomes Star™ for Homelessness. Clinical Scholars Review. 2014;7(1):30-41.
55. Onifade Y. The mental health recovery star. Mental Health & Social Inclusion. 2011;15(2):78-87.
56. Dickens G, Weleminsky J, Onifade Y, Sugarman P. Recovery Star: validating user recovery. Psychiatrist Online. 2012;36(2):45-50.
57. Killaspy H, White S, Taylor TL, King M. Psychometric properties of the mental health recovery star. British Journal of Psychiatry. 2012;201(1):65-70.
58. Downtown Emergency Service Center (DESC). Vulnerability Assessment Tool for Determining Eligibility and Allocating Services and Housing for Homeless Adults.: Downtown Emergency Service Center (DESC). 2009. http://www.desc.org/documents/06.30.2015.DESC.Intro_to_Vulnerability_Assessment_Tool.incl%20VAT%20&%201-page%20validity.pdf. Accessed 10 Aug 2016.
59. Ginzler JA, Monroe-DeVita M. Downtown Emergency Service Center’s Vulnerability Assessment Tool for individuals coping with chronic homelessness: A psychometric analysis: The University of Washington. 2010. http://www.desc.org/documents/DESC%20VAT%20WIMHRT%20final%20report%20031510.pdf. Accessed 01 Feb 2017.
60. Van Brakel WH, Anderson AM, Mutatkar R, Bakirtzief Z, Nicholls PG, Raju M et al. The Participation Scale: measuring a key concept in public health. Disability and rehabilitation. 2006;28(4):193-203.
61. Le Boutillier C, Croucher A. Social inclusion and mental health. British Journal of Occupational Therapy. 2010;73(3):136-9.
